# Prevalence of gram-negative bacteria and their antibiotic resistance in neonatal sepsis in Iran: a systematic review and meta-analysis

**DOI:** 10.1186/s12879-023-08508-1

**Published:** 2023-08-15

**Authors:** Nazila Moftian, Peyman Rezaei-hachesu, Morteza Arab-Zozani, Taha Samad-soltani, Atefeh Esfandiari, Mohammad Saleh Tabib, Kayvan Mirnia

**Affiliations:** 1 Department of Health Information Technology, Faculty of Management and Medical Informatics, Tabriz University of Medical Sciences, Tabriz, Iran; 2 Social Determinants of Health Research Center, Birjand University of Medical Sciences, Birjand, Iran; 3 Department of Health Policy & Management, Faculty of Medicine, Bushehr University of Medical Sciences, Bushehr, Iran; 4 Department of Pediatrics, School of Medicine, Bushehr University of Medical Sciences, Bushehr, Iran; 5 Children Medical Center, Tehran University of Medical Sciences, Tehran Province, Keshavarz Blvd, P94M+85P, Tehran, 14197 33151 Iran

**Keywords:** Neonatal sepsis, Gram-negative bacteria, Antibiotic resistance, Infection diseases, Prevalence rate, Morbidity, Systematic review or meta-analysis

## Abstract

**Background:**

Neonatal sepsis, particularly gram-negative (GN) bacteria-induced, is a significant cause of morbidity and mortality in newborns. Healthcare professionals find this issue challenging because of antibiotic resistance. This study aims to combine findings to identify the prevalence of GN bacteria and their antibiotic resistance in Iranian neonates with sepsis.

**Methods:**

This systematic review followed the Preferred Reporting Items for Systematic Reviews and Meta-Analysis (PRISMA). The literature search was performed through international databases, including (PubMed/MEDLINE, EMBASE, Scopus, and Web of Science), Iranian local databases (Magiran, Iranmedex, Irandoc, Scimed, and SID), and the first 100 records of Google Scholar. Analytical cross-sectional study checklist from the Joanna Briggs Institute (JBI) was used for the quality assessment of included studies. Comprehensive Meta-Analysis Software Version 2 was used to conduct the meta-analysis. The between-study heterogeneity was investigated by I^2^ statistics.

**Results:**

The prevalence of GN bacteria was estimated to be 53.6% [95% CI: 45.9– 61.1: *P* = 0.362] in Iranian neonates with sepsis, based on 31 studies with a sample size of 104,566. klebsiella pneumoniae (K.pneumonia) (23.2% [95% CI: 17.5–30.0, *P* < 0.001]) followed by Escherichia coli (E.coli) (13.5% [95% CI: 9.4–18.9, *P* < 0.001]) were more prevalent among GN bacteria. The highest resistance in K.pneumoniae was observed in Cefixime (80.6%, [95% CI: 56.3–93.1, *P* = 0.018]). E.coli showed greater resistance to Ampicillin (61.8%, [95% CI: 44.2–76.5, *P* = 0.188]. The prevalence of GN bacteria in Iranian neonates with sepsis has a decreasing trend based on the year, as shown by a meta-regression model (*P* < 0.0004).

**Conclusion:**

GN pathogens, particularly K.pneumoniae, and E.coli, are the leading cause of neonatal sepsis in Iran. GN bacteria showed the highest resistance to Third-generation cephalosporin and Aminoglycosides.

**Supplementary Information:**

The online version contains supplementary material available at 10.1186/s12879-023-08508-1.

## Introduction

The neonatal mortality rate is a crucial health indicator. Infections cause almost one-fourth (23%) of neonatal deaths worldwide, with 15% of these deaths resulting from neonatal sepsis [1]. Sepsis is a systemic inflammatory reaction caused by microorganisms invading the bloodstream, leading to extreme symptoms such as fever and shock [2]. Neonatal sepsis is classified into early-onset sepsis (EOS) and late-onset sepsis (LOS). EOS is defined as sepsis within 72 h of birth, and LOS defines as sepsis occurring at or after 72 h of life [3]. Early detection of neonatal sepsis is challenging, so antibiotics are given empirically when sepsis is suspected to prevent severe consequences.

The unnecessary use of broad-spectrum antibiotics in empirical therapy leads to an increase in multidrug-resistant microorganisms in neonatal intensive care units (NICU) and puts a high burden on developing countries. The world health organization (WHO) defines antibiotic resistance as a major public health issue that requires immediate attention [4].

Gram-negative (GN) bacteria-induced neonatal sepsis is a crucial cause of morbidity and mortality in neonates [5]. Neonatal GN sepsis is becoming more prevalent globally, with a concerning rise in multidrug-resistant infections [3, 6]. It has been estimated that 214,000 deaths from neonatal sepsis are attributed to resistant pathogens annually [7]. Sepsis is the fourth leading cause of neonatal mortality in Iran, with an estimated 16% prevalence in hospitalized neonates [8–10]. The high use of empirical and prophylactic antibiotics goes against the recommended therapies [11]. Healthcare professionals face a challenge due to antibiotic resistance. We conducted a systematic review and meta-analysis of published data on gram-negative neonatal sepsis from various regions of Iran due to the increasing evidence of multidrug resistance in neonatal sepsis caused by GN bacteria. The aim was to determine the prevalence of gram-negative bacteria and their antibiotic resistance pattern in neonatal sepsis.

## Materials and methods

The systematic review followed the Preferred reporting items for systematic reviews and meta-analysis (PRISMA) guidelines for systematic reviews and meta-analyses [12]. The review methods were not established prior to the conduct of the review.

### Eligibility criteria

Cross-sectional studies reporting bacterial blood culture and antibiotic resistance/sensitivity testing for neonates with sepsis were included if published in English or Persian language, performed in Iranian hospitals, and used a recognized standard for interpreting antibiotic susceptibility testing (European Committee on Antimicrobial Susceptibility Testing (EUCAST), Clinical and Laboratory Standards Institute (CLSI), British Society for Antimicrobial Chemotherapy). According to the WHO definition, a neonate or newborn infant is a child who is under 28 days old. Any samples over 28 days in age were excluded from the studies. Studies that only reported antibiotic sensitivity were excluded from the analysis. Studies that only reported gram-positive bacteria were excluded. Review studies, letters, case reports, and conference papers were excluded.

### Information sources and search strategy

Four international electronic databases (PubMed/MEDLINE, EMBASE, Scopus, and Web of Science) and five Iranian databases (Magiran, Iranmedex, Irandoc, Scimed, and SID) underwent a broad electronic search. Additionally, we manually searched the first 100 records on Google Scholar. The databases were searched from the beginning up until July 28, 2023. Additionally, the references of included studies were searched for other potentially essential studies. Experts in neonatology and library science were consulted to select the search keywords. The used keywords in this study were as follows: ‘sepsis’, ‘septicemia’, ‘bacteremia’, ‘blood infection’, ‘infant’, ‘newborn’, ‘neonate’, ‘antibiotic resistance’, ‘antimicrobial resistance’, ‘Prevalence’, and their Persian equivalent. Our search was restricted to English and Persian publications. Detailed search strategies for PubMed database available in Supplementary file 1.

### Study selection

All records have been imported to EndNote X8 and duplicates were eliminated. The records were screened by two reviewers, who independently considered inclusion and exclusion criteria based on title and abstract (MST, KM). The full-text of the selected articles was reviewed independently by two different reviewers (PRH, NM). Any disagreement was resolved through discussion among at least three reviewers (KM, MST, NM) until they reached a consensus.

### Data extraction and data items

We used a researcher made data extraction checklist. The data extraction sheet underwent a pilot test on 10 randomly selected articles, followed by revisions and approval by consensus among researchers. The data items collected for every study consisted of author names, publication year, province, duration, hospital type, sample size (categorized by gender), positive culture (categorized by gender), early or late-onset sepsis, pathogen type, and antibiotic resistance. Data extraction was done by two reviewers independently. In case of disagreement, a third author was involved.

### Quality assessment

The quality of the included studies was assessed using the analytical cross-sectional study checklist from the Joanna Briggs Institute (JBI) [13]. The checklist has eight questions that are signed with the answer “Yes”, “No”, and “Unclear”. Articles that scored above 7 were considered high-quality, while those between 4 and 6 were medium-quality, and those below 4 were low-quality. Two reviewers (MN and TSS) conducted the quality assessment and resolved discrepancies through consensus.

### Synthesis of results

The Mantel–Haenszel method was used in performing a meta-analysis with comprehensive meta-analysis (CMA) (Version 2) software. Statistical heterogeneity was evaluated through the calculation of I^2^ statistics. We utilized a fixed or random-effect model with a 95% confidence interval (CI) depending on the level of heterogeneity. In the following of Cochrane criteria if the heterogeneity was ≥ 50 we used the random-effect model. To investigate sources of heterogeneity, sensitivity, and subgroup analyses were conducted, as well as meta-regression models. For each variable, the event rate was determined alongside a 95% CI. Egger's test and funnel plots were used to evaluate the presence of publication bias.

## Results

### Study selection

**Fig. 1:**
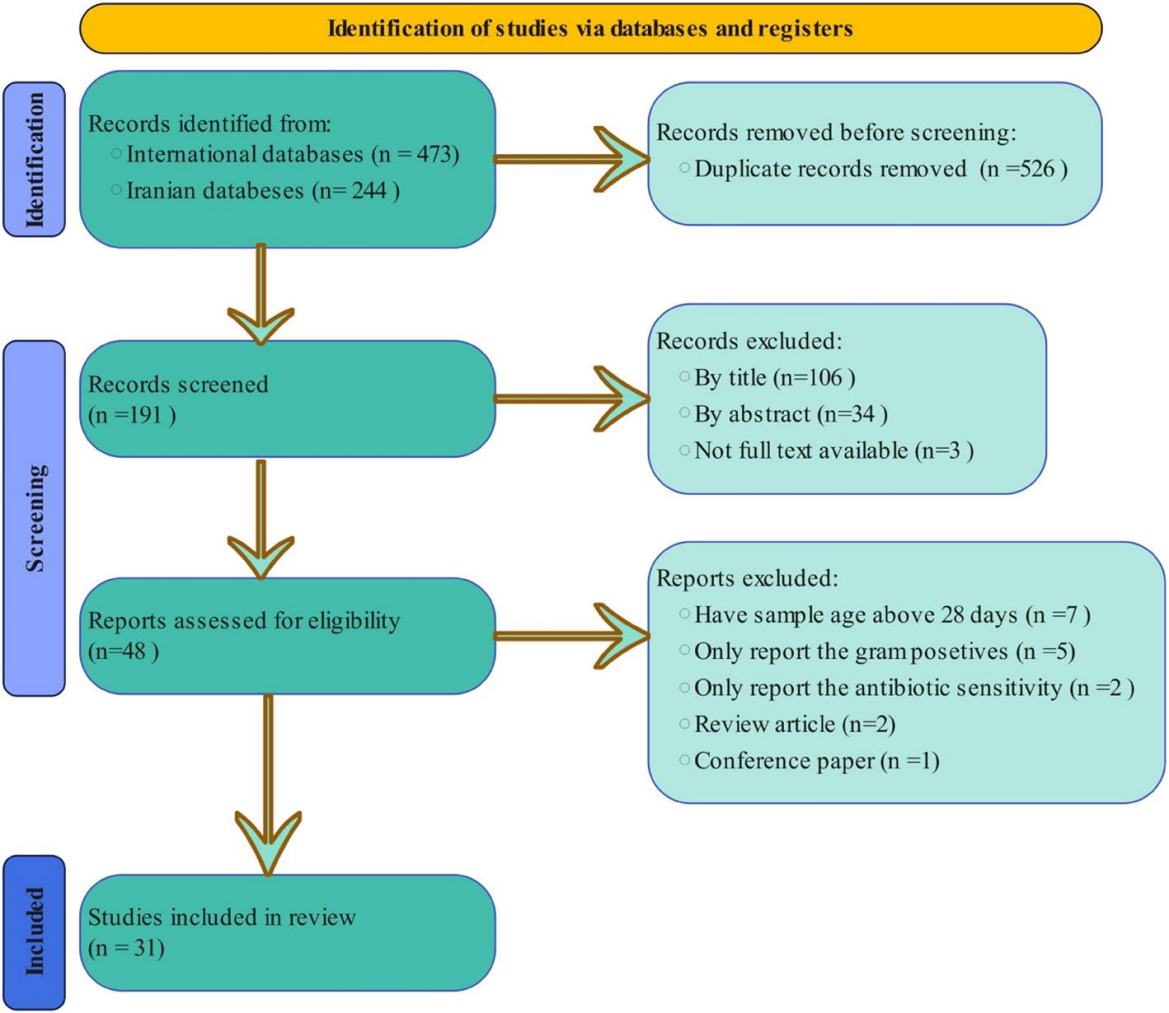
The follow diagram of the literature selection process

Figure 1 displays the flow diagram according to PRISMA guidelines, illustrating the search process and study selection. A total of 717 titles were retrieved from the databases. After removing duplicates, 191 papers were screened by title and abstract for possible inclusion in the study. After applying the eligibility criteria, 48 full-text articles remained for assessment. Based on the exclusion criteria, 17 articles were excluded after the assessment (Age of patients in seven studies was above 28 days, five studies reported just gram-positive bacteria, in two studies only antibiotic sensitivity was reported, two review studies and one study was conference paper). The review included 31 articles [14–44] that met the eligibility criteria.

### Characteristics of the selected studies

The studies that were included were published between 1998 and 2021, with the majority conducted in Iran's Center (*n* = 10) [35–44] based on geographical location, followed by the Northwest (*n* = 5) [30–34], West (*n* = 5) [25–29], North (*n* = 4) [14–17], East (*n* = 4) [21–24], and South (*n* = 3) [18–20]. The duration of the studies varied from six months to 10 years. Of all the studies, 22 were conducted in NICUs of maternity hospitals and nine in NICUs of children's hospitals. Studies assessed 104,566 neonates, among whom 6348 patients had positive blood cultures (6.07% of all cultures). Of all isolates, 69.8% were GN bacteria. Out of 24 studies that report blood cultures based on gender, 2474 males and 1919 females were found to have positive blood cultures. According to Table 1, sepsis was divided into EOS (*n* = 1030) and LOS (*n* = 486) into 12 studies.

**Table 1:** Characteristics of included studies

	Author	Year	Province	Study location	Duration	Sample size		Positive culture		Early or late onset sepsis	
						Male	Female	Male	Female	EOS	LOS
North	Karambin M. et al. [14]	2011	Gilan	Children’s hospital	2 Year	331	280	37	27	5	49
	Fatehi T. et al. [15]	2017	Gilan	-	6 Month	163		16		-	-
	Rafati M.R et al. [16]	2014	Mazandaran	Maternity hospitals	-	63	37	3	17	-	-
	Mozafari A. et al. [17]	2006	Mazandaran	-	1 Year	138	60	11	18	-	-
South	Shahian M. et al. [18]	2010	Fars	Maternity hospitals	30 Month	115	93	54	36	38	52
East	Sedigh Ebrahim H. et al. [19]	2016	Fars	Maternity hospitals	2 Year	491		58	16	-	-
	Rezaei A. et al. [20]	2021	Fars	Maternity hospitals	2 Year	-		250		250	0
	Behmadi H. et al. [21]	2016	Khorasan Razavi	Maternity hospitals	1 Year	1111		53	79	86	46
	Mohammadi N et al. [22]	2007	Khorasan Razavi	Maternity hospitals	9 Month	90	85	18	14	10	22
	Boskabadi H. et al. [23]	2021	Khorasan Razavi	Maternity hospitals	10 Year	5436		268	-	-	10 Year
	Falahi J. et al. [24]	2016	Khorasan Razavi	Maternity hospitals	1 Year	130	240	27		-	-
West	Aletayeb S.M.H. et al. [25]	2011	Khuzestan	Maternity hospitals	54 Month	2500	1200	102	51	99	54
	Monsef A. et al. [26]	2010	Hamedan	Maternity hospitals	2 Year	239	183	60	45	-	-
	Dezfoulimanesh Z et al. [27]	2011	Kermanshah	Maternity hospitals	2 Year	1348	827	63	27	-	-
	Nikkhoo B. et al. [28]	2015	Kurdistan	Maternity hospitals	2 Year	427		13	17	-	-
	Bahmani N. et al. [29]	2021	Kurdistan	Maternity hospitals	1 Year	430		25	16	17	24
Northwest	Ghotaslou R. et al. [30]	2007	East Azerbaijan	Children’s hospital	3 Year	223		119	81	112	88
	Mahallei M. et al. [31]	2018	East Azerbaijan	Children’s hospital	1 Year	838		67		-	-
	Hosseini M. et al. [32]	2019	East Azerbaijan	Maternity hospitals	2 Year	107	67	107	67	-	-
	Gheybi SH. et al. [33]	2008	West Azerbaijan	Maternity hospitals	50 month	2325		142	85	164	63
	Bakhsi khaniki GH., et al. [34]	2011	West Azerbaijan	Maternity hospitals	1 Year	274	128	22	14	-	-
	Bakhsi khaniki GH., et al. [34]	2011	West Azerbaijan	Maternity hospitals	1 Year	274	128	22	14	-	-
Center	Sharif M.R. et al. [35]	2000	Isfahan	Maternity hospitals	1 Year	58	35	46	30	-	-
	Malakan Rad E. et al. [36]	2004	Isfahan	Maternity hospitals	3 Year	218	235	104	32	104	32
	Movahedian AH. et al. [37]	2006	Isfahan	Maternity hospitals	3 Year	1680		79	32	86	25
	Rajabi Z. et al. [38]	2012	Tehran	Children’s hospital	7 Month	70	50	100	20	-	-
	Behjati SH. et al. [39]	1998	Tehran	Children’s hospital	3 Year	204	115	49	79	-	-
	Rabirad N. et al. [40]	2014	Tehran	Children’s hospital	1 Year	11,446		910		-	-
	Marzban A. et al. [41]	2010	Tehran	Children’s hospital	5 Year	2048		207		-	-
	Tehrani F. et al. [42]	2017	Tehran	Maternity hospitals	8 Year	90		90		59	31
	Rajabi Z. et al. [43]	2015	Tehran	Maternity hospitals	7 Month	105		100	20	-	-
	Mahmoudi S. et al. [44]	2017	Tehran	Children’s hospital	6 Year	68,233		1209	1116	-	-

### Assessment quality of articles

Table 2 displays the results of the methodological evaluation of the included studies. The methodological quality of the studies included had a final score range of 5 to 8.

**Table 2:** Methodological evaluation of included studies

	Q1	Q2	Q3	Q4	Q5	Q6	Q7	Q8	Quality point	Quality
Karambin M. et al. [14]	Yes	Yes	Yes	Yes	Yes	Yes	Yes	Yes	8	High
Fatehi T. et al. [15]	Yes	Yes	Yes	Yes	Unclear	Unclear	Yes	Yes	6	Medium
Rafati M.R et al. [16]	Yes	Yes	Yes	Yes	Yes	No	Yes	Yes	7	High
Mozafari A. et al. [17]	Yes	Yes	Unclear	Yes	Unclear	No	Yes	Yes	5	Medium
Shahian M. et al. [18]	No	Yes	Yes	Yes	No	Unclear	Yes	Yes	5	Medium
Sedigh Ebrahim H. et al. [19]	Yes	Yes	Yes	Yes	Yes	Unclear	No	Yes	6	Medium
Rezaei A. et al. [20]	Yes	Yes	Yes	Yes	Yes	Unclear	Yes	Yes	7	High
Behmadi H. et al. [21]	Yes	No	Yes	No	Yes	Unclear	Yes	Yes	5	Medium
Mohammadi N et al. [22]	Yes	Yes	Unclear	Yes	Yes	Yes	Yes	Yes	7	High
Boskabadi H. et al. [23]	Yes	Yes	Yes	Yes	Unclear	No	Yes	Yes	6	Medium
Falahi J. et al. [24]	Yes	Yes	Yes	No	Yes	Unclear	Unclear	Yes	5	Medium
Aletayeb S.M.H. et al. [25]	Yes	Yes	Unclear	Yes	Yes	No	Yes	Yes	6	Medium
Monsef A. et al. [26]	Unclear	Yes	Yes	Yes	Unclear	No	Yes	Yes	5	Medium
Dezfoulimanesh Z et al. [27]	Yes	Yes	Yes	Yes	Yes	Unclear	Yes	Yes	7	High
Nikkhoo B. et al. [28]	Unclear	Yes	Yes	Yes	Unclear	Yes	Yes	Yes	6	Medium
Bahmani N. et al. [29]	Yes	Yes	Unclear	Yes	Yes	Unclear	Yes	Yes	6	Medium
Ghotaslou R. et al. [30]	Yes	Yes	Unclear	Yes	Yes	No	Unclear	Yes	5	Medium
Mahallei M. et al. [31]	Yes	Unclear	No	Yes	Yes	Yes	Yes	Yes	6	Medium
Hosseini M. et al. [32]	Unclear	Yes	Yes	Yes	Yes	Unclear	Yes	Yes	6	Medium
Gheybi SH. et al. [33]	Yes	No	Yes	Yes	Yes	No	Unclear	Yes	5	Medium
Bakhsi khaniki GH., et al. [34]	Yes	Yes	Yes	Yes	Unclear	Unclear	Yes	Yes	6	Medium
Sharif M.R. et al. [35]	Yes	Yes	Unclear	Yes	No	No	Yes	Yes	5	Medium
Malakan Rad E. et al. [36]	Yes	Yes	Unclear	Yes	Unclear	Unclear	Yes	Yes	5	Medium
Movahedian AH. et al. [37]	Yes	Yes	Yes	Yes	Unclear	Yes	Yes	Yes	7	High
Rajabi Z. et al. [38]	Yes	Unclear	No	Yes	Yes	Yes	Unclear	Yes	5	Medium
Behjati SH. et al. [39]	Yes	Yes	Yes	Yes	Unclear	Unclear	Yes	Yes	6	Medium
Rabirad N. et al. [40]	Yes	No	No	Yes	Yes	Unclear	Yes	Yes	5	Medium
Marzban A. et al. [41]	Yes	No	Unclear	No	Yes	Yes	Yes	Yes	5	Medium
Tehrani F. et al. [42]	Yes	Yes	Unclear	Yes	Yes	Unclear	Yes	Yes	6	Medium
Rajabi Z. et al. [43]	Yes	Yes	Yes	Yes	Unclear	Unclear	Yes	Yes	6	Medium
Mahmoudi S. et al. [44]	No	Yes	Unclear	Yes	Yes	Unclear	Yes	Yes	5	Medium
	26	25	18	28	19	7	26	31		

Q1. Were the criteria for inclusion in the sample clearly defined?; Q2. Were the study subjects and the setting described in detail?; Q3. Was the exposure measured in a valid and reliable way?; Q4. Were objective, standard criteria used for measurement of the condition?; Q5. Were confounding factors identified?; Q6. Were strategies to deal with confounding factors stated?; Q7. Were the outcomes measured in a valid and reliable way?; Q8. Was appropriate statistical analysis used?

There were six high-quality studies and 25 medium-quality studies. All studies were included eventually. All studies highlighted Q8 as the most important quality aspect, which confirmed the use of the right statistical analysis. Also, question number 6 which implied “Were strategies to deal with confounding factors stated?” was addressed in seven studies [14, 22, 28, 31, 37, 38, 41].

### Total prevalence of gram-negative bacteria and sensitivity analysis

**Fig. 2:**
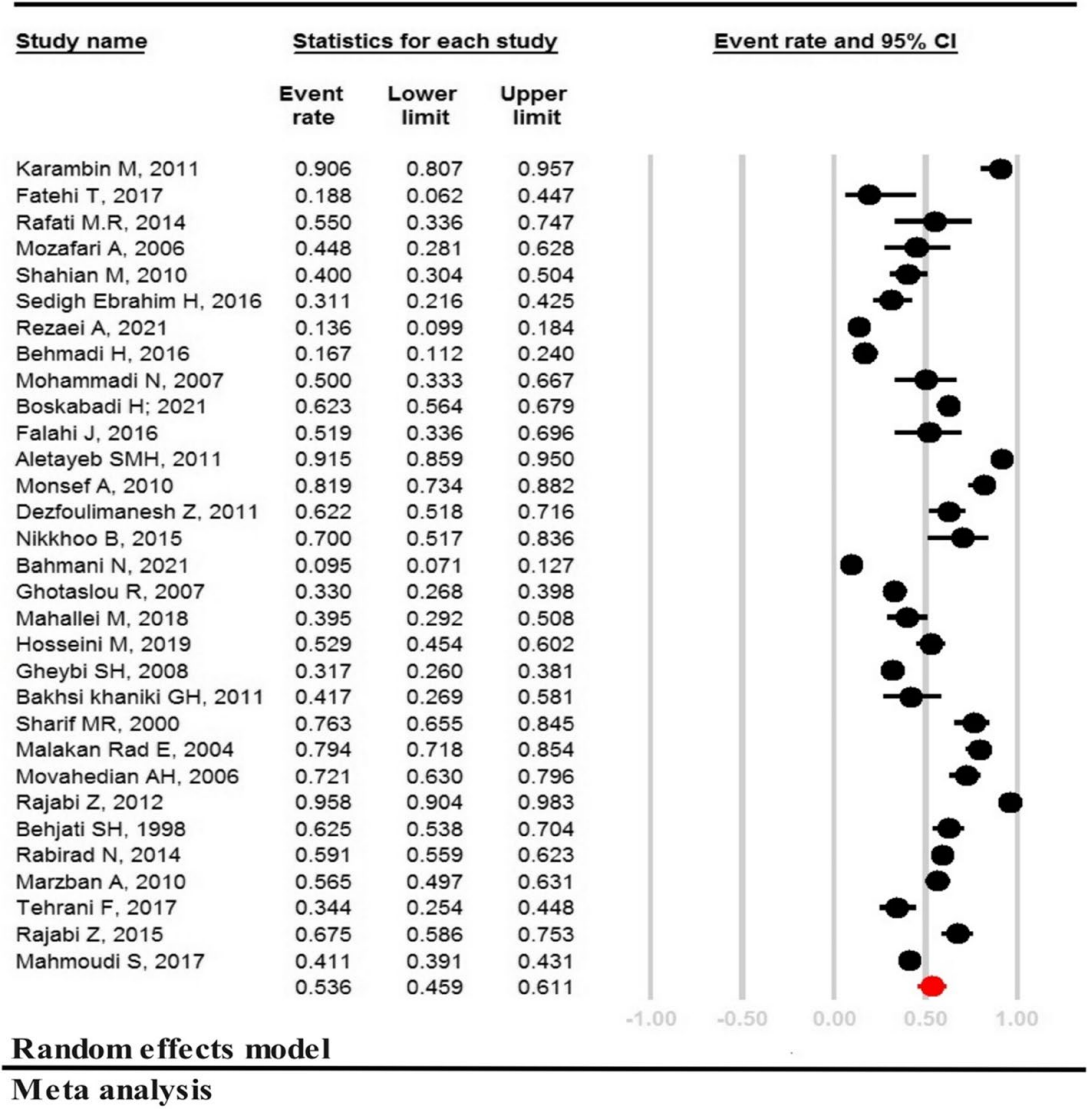
Prevalence of Gram Negative bacteria in neonates with sepsis in Iran

There was a high rate of heterogeneity in the prevalence of GN bacteria (I^2^ = 96.026, *P* < 0.001). According to 31 studies with a sample size of 104,566, GN bacteria in neonates with sepsis was estimated to be 53.6% [95% CI: 45.9– 61.1: *P* = 0.362] (Fig. 2). The studies conducted by Bahmani [29] and Rajabi [38] reported the lowest and highest prevalence of GN bacteria as 9.5% and 95.8%, respectively (Fig. 2).

**Fig. 3:**
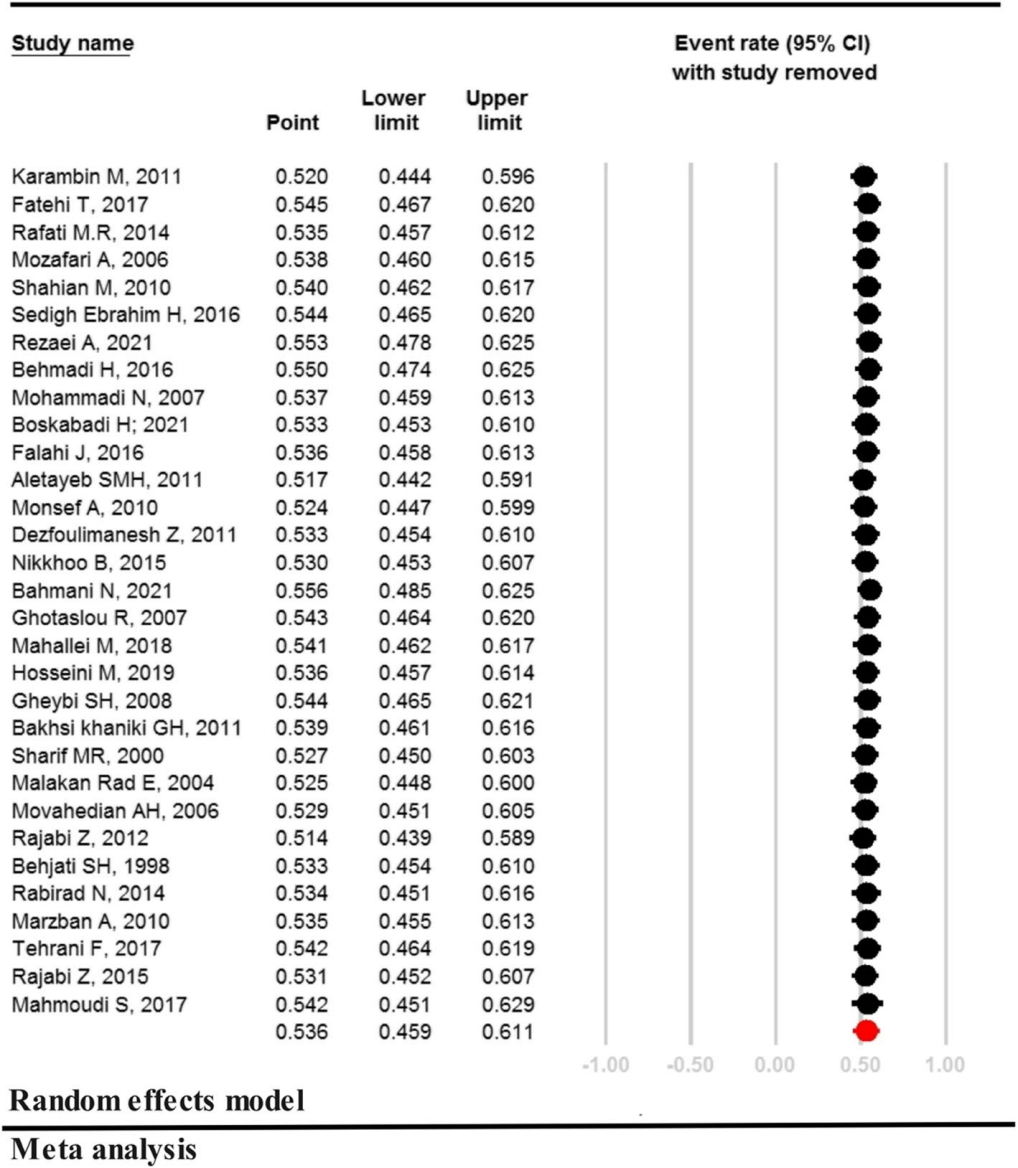
Sensitivity analysis for the prevalence of Gram Negative bacteria in neonates with sepsis in Iran

Sensitivity analysis for the prevalence of GN bacteria in Fig. 3 shows that after removing one study at a time, the result is still robust.

### Subgroup analysis of the prevalence of gram-negative bacteria cause neonatal sepsis based on geographical region

Among GN bacteria that caused neonatal sepsis, Klebsiella pneumoniae (K.pneumonia) (23.2% [95% CI: 17.5–30.0, *P* < 0.001]) followed by Escherichia coli (E.coli) (13.5% [95% CI: 9.4–18.9, *P* < 0.001]) were more prevalent. However, this pattern varied between different regions. As shown in Table 3, in the Center, Northwest, and West of Iran, K.pneumonia had the highest prevalence rate among GN bacteria causing neonatal sepsis (24.6% [95%CI: 16.1–35.6, *P* < 0.001], 17.4% [95%CI: 10.2–28.0, *P* < 0.001], and 19.6% [95%CI: 7.5–42.2, *P* = 0.012], respectively). Also, in the East, North, and South of Iran, E.coli (32.0% [95%CI: 18.0–50.1, *P* < 0.001], 34.4% [95%CI: 21.1–90.5, *P* = 0.009], and 28.8% [95% CI: 4.6–77.2, *P* = 0.403], respectively) had the highest prevalence rate.

**Table 3:** Subgroup analysis for the prevalence of Gram-negative bacteria in neonates with sepsis in Iran

	**Bacteria**	**Studies** **(n)**	**Heterogeneity**		**95%CI**	**Pooled prevalence (%)**	**Model**
			**I**^**2**^	***P*-Value**			
Region							
Center	K.pneumoniae	10	95.312	< 0.001	0.161–0.356	0.246	Random
	E.Coli	6	95.825	< 0.001	0.050–0.173	0.095	Random
	Entrobacter	9	87.132	< 0.001	0.046–0.135	0.079	Random
	P.aeruginosa^a^	7	95.784	< 0.001	0.079–0.239	0.141	Random
	Acinobacter	1	0.000	1.000	0.065–0.101	0.081	Fixed
**Overall**			**96.343**	** < 0.001**	**0.07–0.212**	**0.125**	**Random**
East	K.pneumoniae	4	65.161	0.006	0.083–0.314	0.170	Random
	E.Coli	4	92.931	0.035	0.180–0.501	0.320	Random
	Entrobacter	2	0.000	0.482	0.122–0.193	0.155	Fixed
	P.aeruginosa	1	0.000	1.000	0.010–0.048	0.022	Fixed
	Acinobacter	2	0.000	0.962	0.032–0.083	0.052	Fixed
**Overall**			**91.786**	**0.006**	**0.045–0.259**	**0.114**	**Random**
North	K.pneumoniae	4	90.337	< 0.001	0.0.77–0.539	0.238	Random
	E.Coli	3	91.255	0.001	0.211–0.905	0.344	Random
	Entrobacter	2	65.645	0.054	0.026–0.371	0.110	Random
	P.aeruginosa	2	0.000	0.691	0.012–0.107	0.036	Fixed
	Acinobacter	1	0.000	1.000	0.007–0.282	0.050	Fixed
**Overall**			**90.673**	** < 0.001**	**0.045–0.447**	**0.163**	**Random**
Northwest	K.pneumoniae	5	57.237	0.053	0.102–0.280	0.174	Random
	E.Coli	4	71.373	0.015	0.029–0.121	0.060	Random
	Entrobacter	3	76.726	0.014	0.034–0.163	0.076	Random
	P.aeruginosa	4	54.984	0.083	0.015–0.072	0.033	Random
	Acinobacter	3	94.131	< 0.001	0.021–0.125	0.053	Random
**Overall**			**85.396**	** < 0.001**	**0.035–0.136**	**0.070**	**Random**
South	K.pneumoniae	3	89.000	< 0.001	0.018–0.606	0143	Random
	E.Coli	3	95.260	< 0.001	0.046–0.772	0.288	Random
	Entrobacter	3	96.371	< 0.001	0.019–0.647	0.160	Random
	P.aeruginosa	2	87.867	0.004	0.012–0.794	0.179	Random
	Acinobacter	3	97.163	< 0.001	0.028–0.725	0.215	Random
**Overall**			**93.786**	** < 0.001**	**0.079–0.406**	**0.195**	**Random**
West	K.pneumoniae	4	93.781	< 0.001	0.075–0.422	0.196	Random
	E.Coli	5	93.812	< 0.001	0.081–0.397	0.194	Random
	Entrobacter	4	75.476	0.003	0.058–0.325	0.147	Random
	P.aeruginosa	2	0.000	0.617	0.032–0.091	0.054	Fixed
	Acinobacter	3	90.238	< 0.001	0.031–0.319	0.109	Random
**Overall**			**91.756**	** < 0.001**	**0.079–0.239**	**0.141**	**Random**
Hospital type							
Maternity 's hospital	K.pneumoniae	21	88.401	< 0.001	0.154–0.337	0.203	Random
	E.Coli	20	92.033	< 0.001	0.206–0.288	0.233	Random
	Entrobacter	17	90.020	< 0.001	0.083–0.223	0.139	Random
	P.aeruginosa	13	94.679	< 0.001	0.054–0.189	0.100	Random
	Acinobacter	11	93.967	< 0.001	0.053–0.283	0.117	Random
**Overall**			**91.696**	** < 0.001**	**0.104–0.210**	**0.149**	**Random**
Children’s hospital	K.pneumoniae	9	95.709	< 0.001	0.118–0.332	0.205	Random
	E.Coli	5	96.987	< 0.001	0.030–0.155	0.070	Random
	Entrobacter	7	96.897	< 0.001	0.059–0.217	0.116	Random
	P.aeruginosa	5	87.844	< 0.001	0.026–0.142	0.062	Random
	Acinobacter	2	87.741	0.004	0.011–0.157	0.043	Random
**Overall**			**96.976**	** < 0.001**	**0.049–0.173**	**0.094**	**Random**

^a^Pseudomonas aeruginosa

### Subgroup analysis of the prevalence of Gram-negative bacteria cause neonatal sepsis based on hospital

Hospitals exhibited varying patterns of GN bacteria prevalence. The data in Table 3 shows that E.coli (23.3%, [95% CI: 20.6 -28.8, *P* < 0.001]) and K.pneumonia (20.3%, [95% CI: 15.4–33.7, *P* < 0.001]) were the most common bacteria found in maternity hospitals. While in the children’s hospitals, K.pneumonia (20.5%, [95% CI: 11.8–33.2, *P* < 0.001]) followed by Enterobacter (11.6%, [95% CI: 5.9–21.7, *P* < 0.001]) were more prevalent.

### Prevalence of antibiotic resistance in gram-negative bacteria

**Fig. 4:**
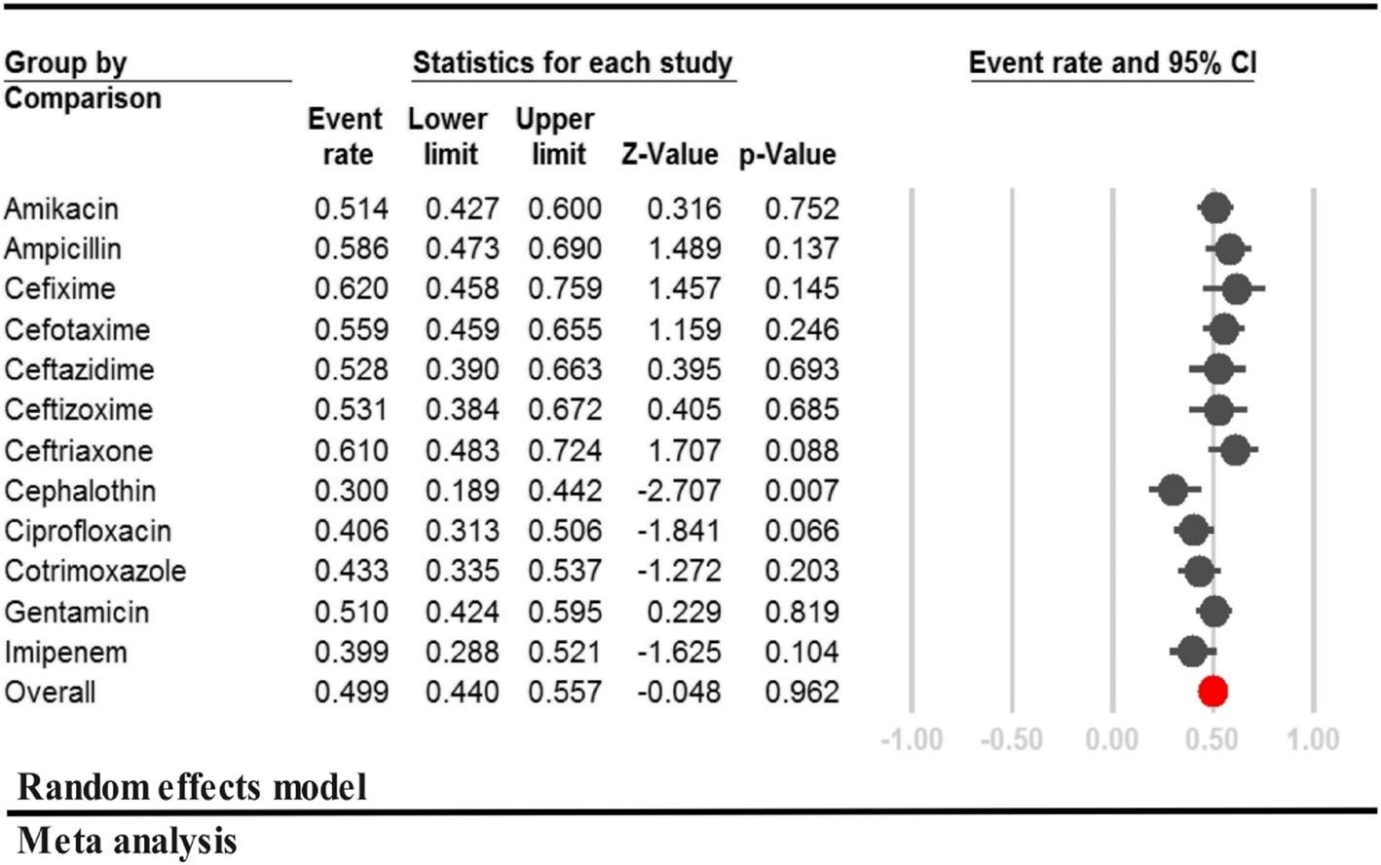
Prevalence of antibiotic resistant in Gram-negative bacteria among neonates with sepsis in Iran

There was a high level of heterogeneity in antibiotic resistance prevalence among GN bacteria (I^2^ = 96.18, *P* < 0.001). Cefixime had the highest resistance rate among third-generation cephalosporins (62.0%, [95% CI: 45.8–75.9, *P* = 0.146]) as shown in Fig. 4. Ampicillin and Amikacin had the highest resistance rates among penicillin and aminoglycosides, respectively (58.6%, [95% CI: 47.3- 69.0, *P* = 0.137] and 51.4%, [95% CI: 42.7–60.0, *P* = 0.616]).

### Subgroup analysis of the prevalence of antimicrobial resistance based on geographic region

**Fig. 5:**
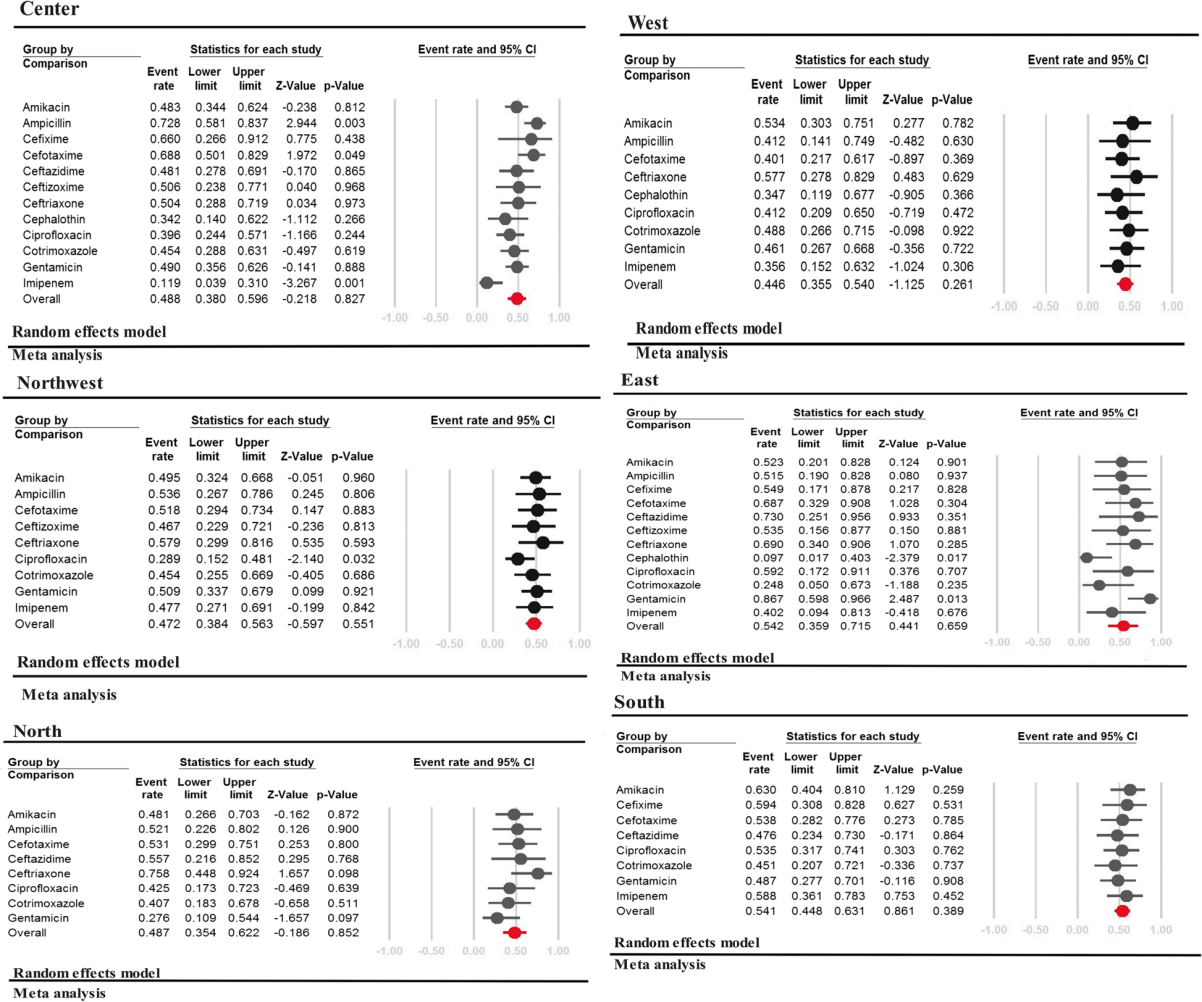
Prevalence of antimicrobial resistance on gram negative bacteria based on geographic region

Figure 5 displays the pattern of antibiotic resistance rate in different regions of Iran. Ampicillin was found to have the highest rate of antibiotic resistance among neonates with sepsis in the Center of Iran (72.8%, [95% CI: 58.1–83.7, *P* = 0.003]). High resistance to Gentamicin (86.7%, [95% CI: 59.8- 96.6, *P* = 0.013]) was observed in the Eastern region of Iran. Ceftriaxone showed the highest resistance rate in the North, Northwest, and West regions (75.8%, [95% CI: 44.8–92.4, *P* = 0.098], 57.9% [95% CI: 29.9–81.6, *P* = 0.593] and 57.7%, [95% CI: 27.8–82.9, *P* = 0.629], respectively). The South of Iran had the highest resistance to Amikacin at 63.0% [95% CI: 40.4–81.0, *P* = 0.117]. Imipenem showed the lowest resistance in the Center of Iran (11.9%, [95% CI: 3.9–31.0, *P* = 0.001]). Both East and West regions exhibited low resistance to Cephalothin (9.7%, [95% CI: 1.6- 41.2, *P* = 0.017] and 34.7%, [95% CI: 11.9–67.7, *P* = 0.366]). Gentamicin showed the lowest resistance rate in the North of Iran (27.6%, [95% CI: 10.9–54.4, *P* = 0.097]). Cotrimoxazole had the lowest resistance in the South (45.1% [95% CI: 20.7–72.1, *P* = 0.751]). Northwest had the lowest resistance rate for Ciprofloxacin (28.9%, [95% CI: 15.2–48.1, *P* = 0.032]).

### Subgroup analysis of the prevalence of antimicrobial resistance based on the type of bacteria

Cefixime was less effective against K.pneumonia, the most resistant GN bacteria causing neonatal sepsis (80.7%, [95% CI: 56.2–93.2, *P* = 0.018]). E.coli was more resistant to Ampicillin (61.7%, [95% CI: 44.3–76.5, *P* = 0.188]), Enterobacter was resistant to Cephalothin (74.2%, [95% CI: 36.6–91.4, *P* = 0.052]) and Acinetobacter was resistant to Cefotaxime (90.0%, [95% CI: [95% CI: 64.7- 97.8, *P* = 0.007]). Pseudomonas aeruginosa (P.aeruginosa) was more resistant to Ceftizoxime (94.7%, [95% CI: 79.5–98.8, *P* < 0.001]). Table 4 displays the antibiotic resistance pattern of two common GN bacteria. Supplementary file 2, Table S1 demonstrates the resistance pattern of other bacteria.

**Table 4:** Subgroup analysis for the antibiotic resistance pattern among two more prevalent gram-negative bacteria

Bactria	Antibiotic	Studies (n)	Heterogeneity		95%CI	Pooled prevalence (%)	Model
			I^2^	*P*-Value			
K.pneumoniae	Amikacin	22	91.391	< 0.001	0.404–0.645	0.526	Random
	Ampicillin	15	95.984	< 0.001	0.467–0.761	0.625	Random
	Cefixime	5	76.380	0.002	0.562–0.932	0.807	Random
	Cefotaxime	17	96.371	< 0.001	0.490–0.758	0.634	Random
	Ceftazidime	8	92.319	< 0.001	0.547–0.874	0.744	Random
	Ceftizoxime	8	92.487	< 0.001	0.423–0.816	0.643	Random
	Ceftriaxone	13	84.418	< 0.001	0.481–0.774	0.640	Random
	Cephalothin	8	94.824	< 0.001	0.522–0.868	0.728	Random
	Ciprofloxacin	15	92.242	< 0.001	0.296–0.592	0.439	Random
	Cotrimoxazole	13	92.790	< 0.001	0.292–0.600	0.440	Random
	Gentamicin	24	91.059	< 0.001	0.513–0.735	0.613	Random
	Imipenem	10	97.304	< 0.001	0.258–0.634	0.454	Random
**Overall**			**94.856**	** < 0.001**	**0.521–0.675**	**0.600**	**Random**
E.Coli	Amikacin	17	95.123	< 0.001	0.332–0.567	0.441	Random
	Ampicillin	10	97.262	< 0.001	0.443–0.765	0.617	Random
	Cefixime	8	78.180	< 0.001	0.377–0.750	0.574	Random
	Cefotaxime	13	94.598	< 0.001	0.358–0.635	0.496	Random
	Ceftazidime	9	92.769	< 0.001	0.315–0.652	0.481	Random
	Ceftizoxime	5	83.306	< 0.001	0.232–0.655	0.431	Random
	Ceftriaxone	6	83.897	< 0.001	0.321–0.730	0.531	Random
	Cephalothin	5	85.061	< 0.001	0.289–0.737	0.517	Random
	Ciprofloxacin	15	91.268	< 0.001	0.316–0.575	0.441	Random
	Cotrimoxazole	14	94.255	< 0.001	0.293–0.557	0.419	Random
	Gentamicin	19	91.375	< 0.001	0.280–0.511	0.389	Random
	Imipenem	10	97.744	< 0.001	0.227–0.527	0.364	Random
**Overall**			**96.213**	** < 0.001**	**0.405–0.527**	**0.466**	**Random**

### Meta-regression

**Fig. 6:**
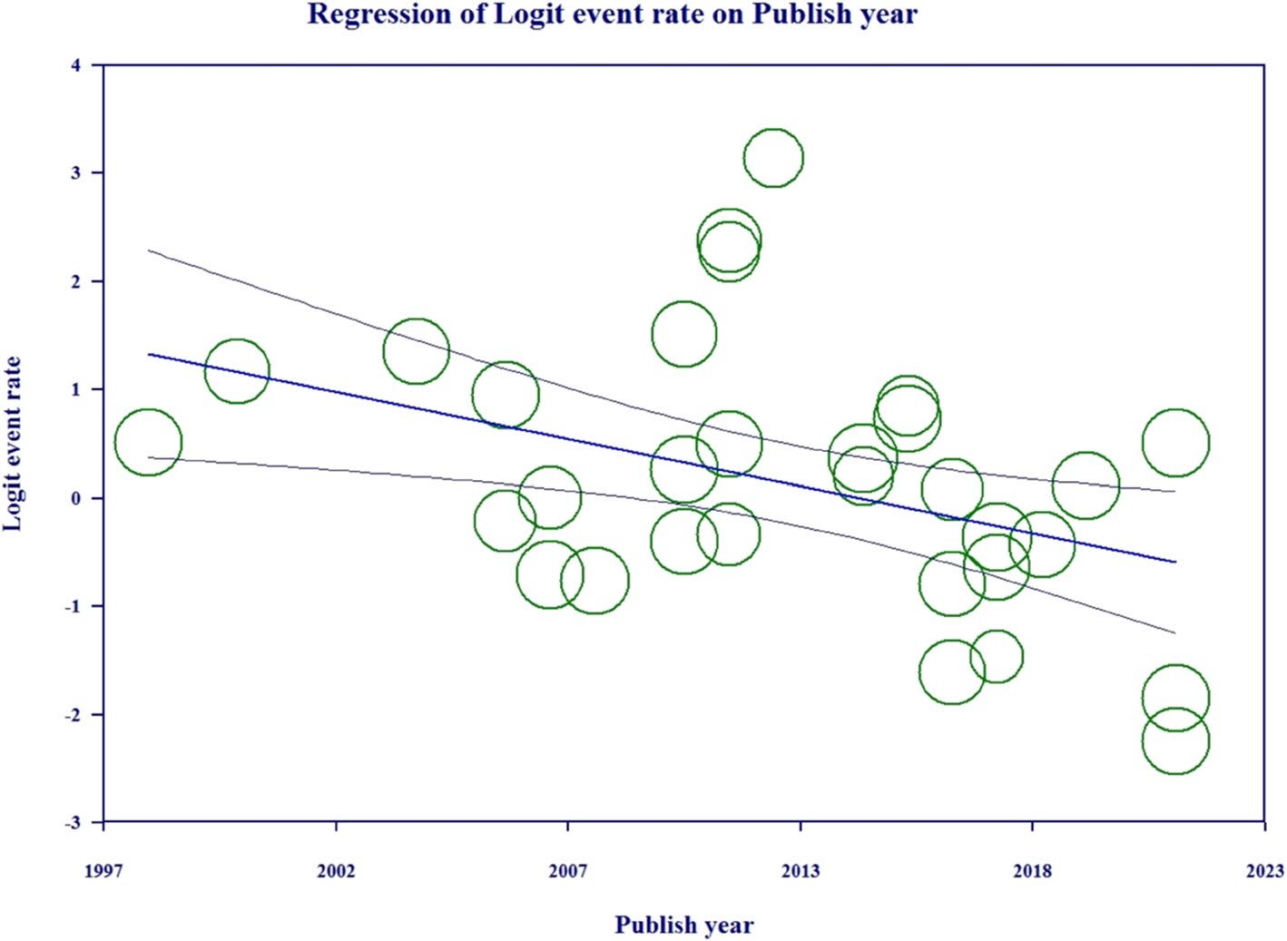
Meta-regression model for the prevalence of gram negative bacteria in neonates with sepsis according to the published year of studies

In Iran, there has been a statistically significant decreasing trend in the prevalence of GN bacteria in neonates with sepsis in recent years, as shown by a meta-regression model that considers the published year of studies (*P* < 0.001) (Fig. 6). The meta-regression model revealed that Ampicillin resistance has been on the rise in recent years in the Center of Iran (*P* < 0.001), while Gentamicin resistance has significantly decreased in the Northwest. The other antibiotics did not exhibit a significant trend (*P* < 0.001).

### Publication bias

**Fig. 7:**
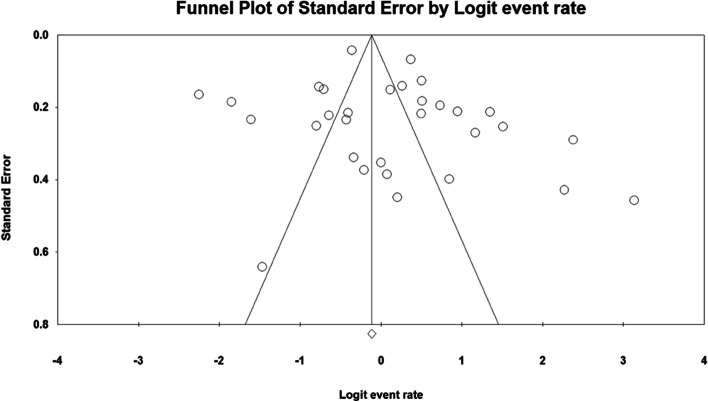
Funnel plot for investigating of publication bias in the included studies

Based on the funnel plot in Fig. 7 and the results of Egger's test, Publication bias was not observed among the included studies (*p* = 0.295).

## Discussion

Our study analyzed the occurrence of GN bacteria and their antibiotic resistance in septic neonates from Iran. Based on the meta-analysis, the occurrence of GN bacteria was found to be 53.6%. Based on the year of studies, the meta-regression model for GN bacteria exhibited a significant decreasing trend. Different studies have reported neonatal sepsis caused by GN agents ranging from 18 to 78% [45–47]. In two systematic reviews conducted in Iran in 2020, Akbarian-Rad et al.[8] reported that Enterobacter (23.04%) and K.pneumonia (17.54%) were common neonatal sepsis GN pathogens after combining 22 articles with a sample size of 14,683. In a review of 17 studies (sample size: 89,472), Akya et al. [9] found that K.pneumonia (24.2%) and P.aeruginosa (16.6%) were the main causative pathogens of neonatal sepsis. The results of our meta-analysis of 31 studies with a total of 104,566 Iranian neonates with sepsis showed that K.pneumonia (23.2%) was the most prevalent GN bacteria, followed by E.coli (13.5%). The advantages of this study over previously published meta-analyses include a larger sample size, the use of cross-sectional studies, and the exclusion of studies with samples over 28 days old. These factors, which were not accounted for in previous meta-analyses, can impact the final evaluation and accuracy of prevalence. Our findings are supported by a 2014 systematic review in resource-limited countries, which demonstrated that in Africa, South-East Asia, and the Middle East, K.pneumonia is often the cause of neonatal sepsis more than other pathogens [48]. Moreover, a systematic review carried out in 2021 in developing countries [49] discovered that K.pneumonia (26.36%) and E.coli (15.30%) were the dominant pathogens responsible for neonatal sepsis. Geographical variation in GN bacteria prevalence was observed among Iranian neonates with sepsis through region-based subgroup analysis. The highest prevalence rate of E.coli was found in the East and North of Iran, at 32.0% and 34.4%, respectively. A systematic review and meta-analysis carried out in Iran in 2019 found that the prevalence rates of urinary tract infection (UTI) and asymptomatic bacteriuria (ASB) in pregnant women were 9.8% and 8.7%, respectively [50]. A higher prevalence of UTI and ASB was observed in the North and East of Iran than in other regions. In addition, E.coli was reported as the predominant microorganism involved in UTI (61.6% [95%CI: 51.6–70.7]) and ASB (63.22% [95%CI: 51.2–73.8]). One reason for the alignment of the results of the current study with that study may be the fact that newborns can get gram-negative bacteria from the vaginal fecal flora of the mother and the environment. Differences in socioeconomic factors, quality healthcare, and racial diversity may explain the variation in neonatal GN bacteria prevalence across geographic regions. The prevalence of GN agents in neonatal with sepsis in Iran, based on the type of hospital, shows that E.coli (23.3%) has the highest prevalence in maternity hospitals and K.pneumonia (20.5%) is more prevalent in children’s hospitals. The rate of prevalence of K.pneumonia in children’s hospitals from 26 to 48% has been reported by various authors [51, 52]. Another study reported K.pneumonia as the most frequently isolated pathogen (32.5%) among extramural admissions [53]. K.pneumonia handles a significant proportion of hospital-acquired infections, such as septicemias [51, 53].

WHO recommends Ampicillin-Gentamicin as the first-line treatment for neonatal sepsis in low- and middle-income countries [54]. Ampicillin and aminoglycoside (Amikacin/Gentamicin) are the primary empirical antibiotics for neonatal sepsis in Iranian NICUs [21]. According to our meta-analysis, nearly 54.0% of GN pathogens that were isolated showed resistance to the WHO-recommended first-line antibiotics. Excessive and irrational use of antibiotics in hospitals may be the cause of high resistance in Iran [11]. The findings of this study align with those of other studies when it comes to levels of resistance to first-line antibiotics [55, 56]. In Africa, Asia, and South America, other reports indicate that 50–80% of neonates have a high resistance rate to commonly used antibiotics, like aminoglycosides, cephalosporins, and ampicillin [57–61]. Depending on the region, the resistance pattern in Iran varied. The increased resistance of GN bacteria to Ampicillin in Iran's Center and its upward trend over the past decade highlights the urgency to re-evaluate the current treatment protocols and implement antibiotic stewardship. The resistance to Gentamicin has lowered in Northwest Iran, perhaps because Amikacin is now the preferred first-line treatment. Local prevention policies and clinical management decisions can be influenced by geographical variations. Ampicillin resistance was observed in both E.coli and K.pneumonia in the current study. Germany, China, and Africa also reported similar findings [48, 62, 63]. A United States report found that 67% of E.coli isolates were resistant to Ampicillin and 17% were resistant to aminoglycosides. Additionally, nearly 10% of the isolates were resistant to both Ampicillin and Gentamicin [64]. Another similar report in 2015–2017 in the United States shows 7.8% of neonatal sepsis caused by E.coli in NICU was resistant to both Ampicillin and Gentamicin [65]. According to previous studies, resistance in E.coli and K.pneumoniae is commonly acquired through plasmidmediated extended-spectrum beta-lactamase (ESBL) production, which has been recognized as a significant threat to public health for the past two decades [66, 67]. ESBL-producing multidrug-resistant bacteria cause infections that are resistant to a variety of beta-lactams, such as third-generation cephalosporins [68]. The effectiveness of third-generation cephalosporins as a second-line treatment is still being debated [63]. Our study found a high level of resistance (57.3%) to third-generation cephalosporins. The reviewed articles in this study were laboratory-based, exploring the resistance of GN bacteria to various types of antibiotic discs. According to the results, Cefixime was found to have the highest resistance in K.pneumoniae. In Iran, Cefixime isn't used as a treatment for neonatal sepsis and Cefotaxime is the second-line treatment for sepsis among third-generation cephalosporins. Acinetobacter showed the highest level of resistance to Cefotaxime. Other studies have reported the high resistance of Acinetobacter to Cefotaxime [69, 70]. Antimicrobial resistance patterns in GN bacteria in Iran make it difficult to choose the right antibiotic for initial empirical therapy. In the NICU, selecting the right empirical antibiotics and treatment duration for suspected sepsis has a lot of variation. Recent studies indicate that implementing NICU-specific antimicrobial stewardship programs (ASP) can significantly reduce the use of inappropriate antibiotics [71, 72]. The use of ASP along with suitable antimicrobial treatments can reduce the negative impact caused by antibiotic resistance in newborns.

Excessive use of broad-spectrum antibiotics in NICUs has led to a serious problem of infections caused by multidrug-resistant GN bacteria in some developing countries. Developed countries face this problem with less severity. The occurrence of multidrug-resistant GN bacteria in the present study is akin to that of China and India [63, 73]. Multidrug resistance was found in more than 50% of GN bloodstream isolates in the present study. This level of resistance highlights the significance of GN multidrug resistance in Iranian neonates. Improving infection control strategies should be prioritized. The essential method for preventing GN multidrug resistance colonization and infection is to restrict horizontal transmission. Infection control measures, such as proper hand hygiene, suitable gloving, disinfection, decontamination, and sterilization practices, should be taken seriously. It is important to prevent unit overcrowding and understaffing. NICU-specific ASPs play a crucial role in reducing resistance. Neonatal ESBL bacterial sepsis incidence can be reduced by limiting cephalosporin. Nevertheless, an important challenge is to minimize the use of third-generation cephalosporins and carbapenems. Additional clinical research is urgently required to address these challenges.

In this meta-analysis, most studies did not differentiate between EOS or LOS cases in sepsis. Unfortunately, grouping by sepsis type for analysis was not feasible. The neonates were not classified based on gender, so a detailed analysis could not be conducted. Another limitation of this study was the uneven distribution of samples across the study regions.

The study's findings are crucial for WHO's antibiotic recommendations for neonatal sepsis. Many neonates may not receive sufficient coverage from common first-line and second-line antibiotics. Therefore, these findings can aid in the creation of NICU-specific antibiotic use guidelines.

## Conclusion

The study emphasizes that K.pneumoniae and E.coli are the most frequent gram-negative pathogens that cause neonatal sepsis in Iran. The distribution of sepsis-causative pathogens differs among hospitals and regions, as shown in this systematic review. GN bacteria showed the greatest resistance to third-generation cephalosporin and aminoglycosides. Neonatologists in Iranian hospitals should carefully discuss this alarming result and consider changing the treatment regimen if needed.

### Supplementary Information

**Additional file 1. **Pubmed search strategy. 

**Additional file 2. **Subgroup analysis for the antibiotic resistance pattern among gram-negative bacteria in Iranian neonates with sepsis. 

## Data Availability

The datasets generated during and/or analyzed during the current study are available from the corresponding author (Email: kayvanmirnia@yahoo.com) and first author (Email: nazila.moftian@gmail.com) on reasonable request.

## Abbreviations

ASB: Asymptomatic bacteriuria
ASP: Antimicrobial stewardship programs
CI: Confidence interval
CLSI: Clinical and Laboratory Standards Institute
CMA: Comprehensive meta-analysis
EOS: Early-onset sepsis
E.coli: Escherichia coli
ESBL: Extended-spectrum beta-lactamase
EUCAST: European Committee on Antimicrobial Susceptibility Testing
GN: Gram-negative
JBI: Joanna briggs institute
K.pneumoniae: Klebsiella pneumoniae
LOS: Late-onset sepsis
NICU: Neonatal intensive care units
PRISMA: Preferred reporting items for systematic reviews and meta-analysis
P.aeruginosa: Pseudomonas aeruginosa
UTI: Urinary tract infection
WHO: World health organization
