# Supplementary material for: Prevalence of gram-negative bacteria and their antibiotic resistance in neonatal sepsis in Iran: a systematic review and meta-analysis
Source: BMC Infect Dis. 2023 Aug 15;23:534. doi: 10.1186/s12879-023-08508-1 (PMC10426195; doi:10.1186/s12879-023-08508-1)
Supplement: Supplementary file 1 — Additional file 1. Pubmed search strategy. [file 12879_2023_8508_MOESM1_ESM.docx]

**Supplementary file 1: Pubmed search strategy**

(sepsis[All Fields] OR septic*[All Fields] OR bacteremia[All Fields] OR “bacteremia"[Mesh] OR septicaemia[All Fields] OR “blood infection”[ All Fields] OR "Sepsis"[Mesh] OR prevalence[All fields]) AND (sensitivity[All Fields] OR susceptibility[All Fields] OR "antimicrobial susceptibility"[ All Fields] OR “antibiotic susceptibility”[All Fields] OR "Microbial sensitivity Tests"[MeSH] OR resistance[All Fields] OR resistant*[ All Fields] OR "Drug Resistance"[Mesh]) AND (newborn*[All Fields] OR "new born"[All Fields] OR "new borns"[All Fields] OR "newly born"[All Fields] OR infant*[All Fields] OR infancy[All Fields] OR neonate[All Fields] OR neonates[All Fields] OR “Infant, Newborn”[Mesh]) AND (antibiotic[All Fields] OR antimicrobial[All Fields] OR anti-microbial[All Fields] OR antibacterial[All Fields] OR anti-bacterial[All Fields] OR drug[All Fields]) AND (iran[All Fields] OR iranian[All Fields] OR “iran"[Mesh])
