# Supplementary material for: Prevalence of gram-negative bacteria and their antibiotic resistance in neonatal sepsis in Iran: a systematic review and meta-analysis
Source: BMC Infect Dis. 2023 Aug 15;23:534. doi: 10.1186/s12879-023-08508-1 (PMC10426195; doi:10.1186/s12879-023-08508-1)
Supplement: Supplementary file 2 — Additional file 2. Subgroup analysis for the antibiotic resistance pattern among gram-negative bacteria in Iranian neonates with sepsis. [file 12879_2023_8508_MOESM2_ESM.docx]

Supplementary file 2: Subgroup analysis for the antibiotic resistance pattern among gram-negative bacteria in Iranian neonates with sepsis

Table S1. Subgroup analysis for the antibiotic resistance pattern among gram-negative bacteria

| Bactria | Antibiotic | Studies  (n) | Heterogeneity | | 95%CI | Pooled prevalence (%) | Model |
| --- | --- | --- | --- | --- | --- | --- | --- |
|  |  |  | I^2^ | P-Value |  |  |  |
|  |  |  |  |  |  |  |  |
| Entrobacter | Amikacin | 17 | 95.227 | <0.001 | 0.485-0.789 | 0.652 | Random |
|  | Ampicillin | 8 | 97.765 | <0.001 | 0.332-0.763 | 0.558 | Random |
|  | Cefixime | 5 | 41.648 | 0.144 | 0.558-0.770 | 0.673 | Fixed |
|  | Cefotaxime | 9 | 76.066 | <0.001 | 0.303-0.716 | 0.511 | Random |
|  | Ceftazidime | 5 | 72.522 | 0.006 | 0.253-0.789 | 0.537 | Random |
|  | Ceftizoxime | 4 | 93.863 | <0.001 | 0.360-0.898 | 0.689 | Random |
|  | Ceftriaxone | 6 | 85.663 | <0.001 | 0.278-0.773 | 0.533 | Random |
|  | Cephalothin | 5 | 92.011 | <0.001 | 0.366-0.914 | 0.742 | Random |
|  | Ciprofloxacin | 6 | 95.146 | <0.001 | 0.496-0.895 | 0.713 | Random |
|  | Cotrimoxazole | 10 | 96.293 | <0.001 | 0.470-0.836 | 0.680 | Random |
|  | Gentamicin | 16 | 93.963 | <0.001 | 0.381-0.706 | 0.549 | Random |
|  | Imipenem | 7 | 98.363 | <0.001 | 0.246-0.711 | 0.472 | Random |
| ***Overall*** |  |  | ***96.152*** | ***<0.001*** | ***0.522-0.688*** | ***0.608*** | ***Random*** |
| Acinobacter | Amikacin | 8 | 97.345 | <0.001 | 0.274-0.755 | 0.533 | Random |
|  | Ampicillin | 4 | 97.278 | <0.001 | 0.123-0.754 | 0.396 | Random |
|  | Cefotaxime | 5 | 89.117 | <0.001 | 0.647-0.978 | 0.900 | Random |
|  | Ceftazidime | 4 | 81.741 | 0.001 | 0.310-0.919 | 0.693 | Random |
|  | Ceftizoxime | 3 | 91.204 | <0.001 | 0.272-0.960 | 0.749 | Random |
|  | Ciprofloxacin | 7 | 95.162 | <0.001 | 0.249-0.799 | 0.534 | Random |
|  | Cotrimoxazole | 2 | 77.912 | 0.033 | 0.079-0.863 | 0.424 | Random |
|  | Gentamicin | 8 | 93.171 | <0.001 | 0.259-0.773 | 0.522 | Random |
|  | Imipenem | 6 | 90.745 | <0.001 | 0.157-0.716 | 0.406 | Random |
| ***Overall*** |  |  | ***96.062*** | ***<0.001*** | ***0.463-0.837*** | ***0.678*** | ***Random*** |
| Pseudomonas aeruginosa | Amikacin | 13 | 93.467 | <0.001 | 0.267-0.534 | 0.393 | Random |
|  | Ampicillin | 5 | 97.396 | <0.001 | 0.313-0.749 | 0.539 | Random |
|  | Cefotaxime | 8 | 91.620 | <0.001 | 0.419-0.809 | 0.636 | Random |
|  | Ceftazidime | 4 | 95.968 | <0.001 | 0.273-0.755 | 0.519 | Random |
|  | Ceftizoxime | 3 | 68.604 | 0.041 | 0.795-0.988 | 0.947 | Random |
|  | Ceftriaxone | 4 | 54.892 | 0.084 | 0.743-0.974 | 0.912 | Random |
|  | Cephalothin | 2 | 90.571 | 0.001 | 0.112-0.821 | 0.433 | Random |
|  | Ciprofloxacin | 3 | 0.000 | 0.541 | 0.197-0.259 | 0.227 | Fixed |
|  | Cotrimoxazole | 5 | 94.169 | <0.001 | 0.505-0.917 | 0.770 | Random |
|  | Gentamicin | 12 | 94.016 | <0.001 | 0.345-0.630 | 0.487 | Random |
|  | Imipenem | 4 | 98.253 | <0.001 | 0.265-0.715 | 0.487 | Random |
| ***Overall*** |  |  | ***95.812*** | ***<0.001*** | ***0.438-0.737*** | ***0.596*** | ***Random*** |
